# Leukocyte Platelet‐Rich Plasma‐Derived Exosomes Restrained Macrophages Viability and Induced Apoptosis, NO Generation, and M1 Polarization

**DOI:** 10.1002/iid3.70064

**Published:** 2024-11-15

**Authors:** Xiong Li, Feifei Guo, Jiehua Deng, Jiasong Li, Jie Zhang, Ming Fu, Hui Fan

**Affiliations:** ^1^ Department of Plastic and Aesthetic Surgery The Second Affiliated Hospital of Guilin Medical University Guilin China; ^2^ Department of Health Management Centre The Second Affiliated Hospital of Guilin Medical University Guilin China; ^3^ Department of Otolaryngology The Second Affiliated Hospital of Guilin Medical University Guilin China

**Keywords:** apoptosis, chronic refractory wounds, L‐PRP, macrophages, NO, polarization, P‐PRP

## Abstract

**Background:**

Chronic refractory wounds refer to wounds that cannot be repaired timely. Platelet‐rich plasma (PRP) has significant potential in chronic wound healing therapy. The exosomes isolated from PRP were proved to exhibit more effectiveness than PRP. However, the therapeutic potential of exosomes from PRP on chronic refractory wounds remained elusive. Hence, this study aimed to clarify the action of exosomes from PRP on chronic refractory wounds by evaluating the response of macrophages to exosomes.

**Methods:**

Pure platelet‐rich plasma (P‐PRP) and leukocyte platelet‐rich plasma (L‐PRP) were prepared from the fasting venous blood of healthy volunteers. Exosomes were extracted from P‐PRP and L‐PRP using ultracentrifugation and identified by transmission electron microscopy (TEM), nanoparticle tracking analysis (NTA), and western blot. Macrophages were obtained by inducing THP‐1 cells with phorbol‐12‐myristate‐13 acetate (PMA). The internalization of exosomes into macrophages was observed utilizing confocal laser scanning microscopy after being labeled with PKH67. Cell viability was determined by CCK‐8 assay. Cell apoptosis was measured utilizing a flow cytometer. The polarization status of M1 and M2 macrophages were evaluated by detecting their markers. Nitric oxide (NO) detection was conducted using the commercial kit.

**Results:**

Exosomes from P‐PRP and L‐PRP were absorbed by macrophages. Exosomes from L‐PRP restrained viability and induced apoptosis of macrophages. Besides, exosomes from P‐PRP promoted M2 polarization, and exosomes from L‐PRP promoted M1 polarization. Furthermore, exosomes from L‐PRP promoted NO generation of macrophages.

**Conclusion:**

Exosomes from L‐PRP restrained viability, induced apoptosis and NO generation of macrophages, and promoted M1 polarization, while exosomes from P‐PRP increased M2 polarization. The exosomes from L‐PRP presented a more effective effect on macrophages than that from P‐PRP, making it a promising strategy for chronic refractory wound management.

## Graphical abstract

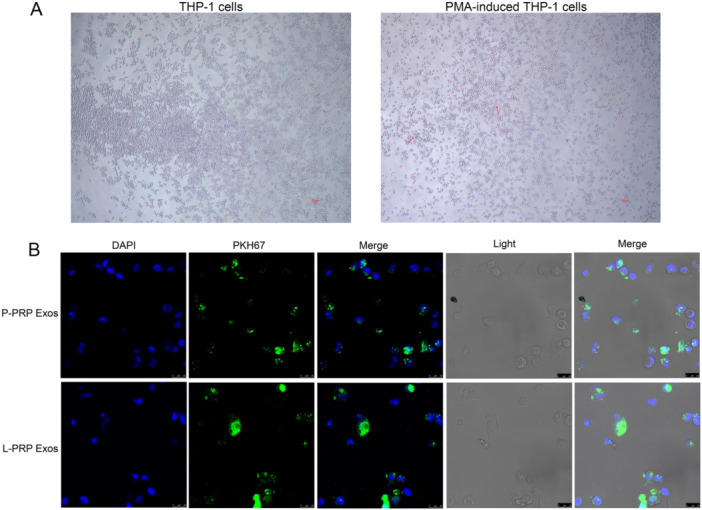

## 1 Introduction

Chronic refractory wounds typically refer to wounds that cannot generally heal within the expected time [1]. Chronic refractory wounds are a multifactorial process caused by various underlying conditions, such as inflammation, diabetes, chronic disease, venous or arterial disease, infection, malnutrition, senescence, local pressure, and edema [1, 2]. It is accepted that aged adults are more susceptible to suffering from chronic refractory wounds than younger people [3]. In clinical treatment, chronic wounds have a high incidence rate, which can be divided into venous ulcer, arterial ulcer, pressure ulcer, and diabetes ulcer [4]. Among these classifications, venous ulcers are the most common type of wounds. Traditional treatment methods for wounds include debridement, wound bed preparation, saline dressings, and infection management [5]. Usually, symptomatic treatment is of no use in improving local blood circulation or reducing local inflammation. Therefore, most patients suffer from long‐term treatment and chronic pain, which reduces their quality of life and increases their economic burden [6, 7]. With the high incidence rate of chronic wounds, the global medical system bears a heavy economic burden, which leads to a high risk of fatal death, amputation and death [8]. Therefore, exploring effective strategies for chronic refractory wound management is urgent.

In histology, the local tissue of a chronic wound shows extensive neutrophil infiltration, leading to excessive inflammation around the wound, and further recruitment of reactive oxygen species and destructive enzymes to continue this cycle [9]. Chronic and excessive inflammation can inhibit wound healing. Hence, microenvironments composed of multiple immune cells are vital for healing human skin wounds [10]. The injured skin needs to activate the immune response at the beginning and end of wound healing, characterized by an extensive recruitment of immune cells [11]. Among these immune cells, macrophages exerted a predominant function in tissue remodeling [12, 13]. Macrophages are recruited to the wound site primarily from the peripheral blood shortly after wound development [14]. M1 macrophages infiltrate the wound area at 1–2 days of injury and are further polarized to M2 macrophages at 3 days after injury, which is necessary for wound healing [15]. Blocking M1–M2 polarization is strongly associated with delayed wound healing [15]. For instance, Limido et al. [16] demonstrated that nanofat could accelerate and improve angiogenesis, lymphatic drainage and healing of full‐layer skin wounds in mice by promoting the transformation of M1‐ to M2‐polarized macrophages. Another study found that the administration of ceAF promoted M2 macrophage polarization and inflammatory response in the wound tissues to attenuate delayed wound healing [17]. Therefore, targeting macrophage polarization may be an effective strategy for managing chronic wound healing.

Platelet‐rich plasma (PRP) is an autologous blood product with a high platelet concentration [18, 19]. Platelets in PRP can produce growth factors and regulatory proteins after activation, critical for cell proliferation and differentiation [20, 21]. According to the total leukocytes within PRP, PRP can be divided into leukocyte‐rich PRP (L‐PRP) and pure platelet‐rich plasma (P‐PRP) [22, 23]. Recently, PRP enhanced macrophage infiltration into tissues in tendon repair, and was proven to improve the healing of refractory wounds, demonstrating enormous potential for wound repair [24]. When PRP supernatants were added to the macrophage culture medium, they could inhibit M1 polarization of monocyte‐derived macrophages, and promote the polarization of monocyte‐derived M1 macrophages to M2 macrophages [25]. It has been reported that L‐PRP could specifically promote the polarization to M2a macrophages, which were mainly associated with anti‐inflammatory activity, thereby participating in injured tissue repairing [26]. Nishio et al. [27] illustrated that the local application of L‐PRP in injured tissues could facilitate recruitment of macrophages from peripheral tissues and blood, as well as L‐PRP mainly enhance the action of M1 macrophages, which indicated that acute infiltration and subsequent recruitment of inflammatory cells may stimulate tissue repair processes more quickly. Furthermore, increasing evidence revealed that the exosomes isolated from PRP exhibited more efficient application potential [28, 29]. For instance, Esmaeilzadeh et al. [30] reported exosomes from PRP had the potential to promote wound healing as they enhance neovascularization and cell migration, and reduce inflammation and scar formation. However, the therapeutic potential of exosomes from PRP on chronic refractory wounds remained elusive.

Hence, this study aimed to clarify the action of exosomes from PRP on chronic refractory wounds by evaluating the response of macrophages to exosomes.

## 2 Materials and Methods

### 2.1 Preparation of P‐PRP and L‐PRP

Fasting venous blood was collected from two male healthy volunteers (age 25 and 37) in the Department of Blood Transfusion, the Second Affiliated Hospital of Guilin Medical College after signing informed consent. The P‐PRP and L‐PRP were prepared using the PRP preparation kits (VEGO, China) and based on the described methods [31]. The concentrations of platelets and WBCs were determined by a blood analyzer. The collected P‐PRP and L‐PRP were preserved in −80°C. The study was approved by the Ethics Committee of the Second Affiliated Hospital of Guilin Medical College.

### 2.2 Isolation and Identification of Exosomes

The P‐PRP and L‐PRP samples were centrifuged at 500*g* for 10 min at 4°C. Then, the supernatant was centrifuged at 2000*g* for 30 min, and the supernatant was collected for another 10,000*g* for 30 min using a centrifuge (5810R, Eppendorf, Hamburg, Germany). Afterward, the supernatant was harvested to a high‐speed centrifuge tube, and the volume was replenished with sterile PBS to 20 mL, and then was centrifuged at 120,000*g* for 70 min at 4°C using a high‐speed centrifuge (Optima XPN‐100, Beckman, Brea, CA, USA) to obtain high‐purity exosomes. The harvested exosomes were identified by CD63, TSG101, and CD9 markers using Western blot. The morphology and size distribution were detected utilizing transmission electron microscopy (TEM) (JOEL Ltd, Japan) and nanoparticle tracking analysis (NTA) (Malvern, UK), respectively.

### 2.3 Cell Culture

THP‐1 cells were obtained from the Shanghai Institute of Cell Biology, Chinese Academy of Sciences (Shanghai, China). All cells were maintained in RPMI 1640 medium with 10% FBS and 1% streptomycin/penicillin (Gibco, Waltham, MA, USA) at 37°C with 5% CO_2_. In indicated assays, THP‐1 cells were incubated with 200 ng/mL phorbol‐12‐myristate‐13 acetate (PMA) to induce differentiation.

### 2.4 Determining the Cellular Uptake of Exosomes

Exosomes were incubated with PKH67 (Thermo Fisher, USA) for 5 min at room temperature, and 1% BSA was used to terminate staining. The free dye was removed, and PKH67‐stained exosomes were collected by centrifugation at 12,000*g* for 90 min.

THP‐1 cells were harvested, seeded in 24‐well plates, and induced into macrophages using PMA. Cells were then cultured in RPMI 1640 medium without serum. After 24 h, cells were incubated with 10 μL of PKH67‐stained exosomes for 24 h. Afterward cells were fixed in 4% paraformaldehyde, treated with 0.1% TritonX‐100, blocked with 3% BSA, and stained with Mounting Medium containing DAPI. Finally, cells were observed and photographed under confocal laser scanning microscopy.

### 2.5 Cell Counting Kit‐8 (CCK‐8) Assay

After THP‐1 cells were collected, cells were added to RPMI 1640 medium with 10% exosome‐free serum and 200 ng/mL PMA. Afterward cells were seeded into 96‐well plates (1 × 10^4^ cells/well) for 24 h cultivation. Then, cells were mixed with different concentrations of exosomes (final concentrations of 5, 10, 20 and 50 μg/mL) for incubation, and the control cells were PMA‐induced THP‐1 cells treated with PBS. At 24, 48, and 72 h, 10 μL CCK‐8 (Beyotime, Shanghai, China) was added to each well and treated for 1 h. Optical density values at wavelength 450 nm were tested by a microplate reader (Bio‐Rad, USA).

### 2.6 Cell Apoptosis

Cell apoptosis was evaluated utilizing Annexin V‐FITC Apoptosis Detection Kit (BD Biosciences, CA, USA). After THP‐1 cells were collected, cells were added to RPMI 1640 medium with 10% exosome‐free serum and 200 ng/mL PMA. Cells were seeded into 24‐well plates (1 × 10^5^ cells/well) for 24 h cultivation. Then, cells were mixed with exosomes (final concentration: 20 μg/mL) for 72 h incubation. After that, THP‐1 cells (1 × 10^5^) were harvested and resuspended in the binding buffer. Then, cells were dyed with 5 μL Annexin V‐FITC and 5 μL propidium iodide (PI) for 15 min without light at room temperature. The apoptotic cells were determined by the FACSCalibur flow cytometer (BD Biosciences, USA).

### 2.7 Western blot

Total proteins were collected using RIPA and quantitated by the BCA method. Proteins were run on the SDS‐PAGE and transferred to PVDF membranes. After hindered in 5% skim milk, the membranes were probed to anti‐TSG101 (1:1000, 28283‐1‐AP), anti‐CD63 (1:1000, 25682‐1‐AP), anti‐CD9 (1:1000, 20597‐1‐AP), anti‐CD206 (1:1000, 18704‐1‐AP), anti‐TGF‐β1 (1:500, sc‐130348), anti‐IL‐1β (1:1000, AF5103), anti‐TNF‐α (1:1000, 60291‐1‐Ig), and GAPDH (1:10000, 60004‐1‐Ig) for 12 h and treated with second antibody IgG. All primary antibodies were acquired from Proteintech (Proteintech, Wuhan, China) except for TGP‐β1 (Santa Cruz, CA, USA) and IL‐1β (Affinity, Jiangsu, China). The bands were detected utilizing an ECL system (Bio‐Rad, USA) and analyzed using Image J.

### 2.8 Quantitative Real‐Time Polymerase Chain Reaction (qRT‐PCR)

Total RNAs were collected using TransZol Up and utilized to generate cDNA by the First‐Strand cDNA Synthesis SuperMix for qPCR (TransGen Biotech, Beijing, China). Following the supplier's protocol, the qPCR assay was carried out using the PerfectStart Green qPCR SuperMix (TransGen Biotech, Beijing, China). Primers used in this study are listed in Table 1. The relative expression of genes was assessed using the $2^{‐∆∆C_{t}}$ via normalizing to GAPDH.

**Table 1:** Primer sequences used in the study.

Primers	Direction	Sequence (5′–3′)
IL6	Forward	CCTGAACCTTCCAAAGATGGC
IL6	Reverse	TTCACCAGGCAAGTCTCCTCA
IL‐1β	Forward	ATGATGGCTTATTACAGTGGCAA
IL‐1β	Reverse	GTCGGAGATTCGTAGCTGGA
TNF‐α	Forward	GAGGCCAAGCCCTGGTATG
TNF‐α	Reverse	CGGGCCGATTGATCTCAGC
CD206	Forward	TCCGGGTGCTGTTCTCCTA
CD206	Reverse	CCAGTCTGTTTTTGATGGCACT
Arginase1	Forward	GTGGAAACTTGCATGGACAAC
Arginase1	Reverse	AATCCTGGCACATCGGGAATC
CD163	Forward	TTTGTCAACTTGAGTCCCTTCAC
CD163	Reverse	TCCCGCTACACTTGTTTTCAC
CD86	Forward	CTGCTCATCTATACACGGTTACC
CD86	Reverse	GGAAACGTCGTACAGTTCTGTG
iNOS	Forward	TTCAGTATCACAACCTCAGCAAG
iNOS	Reverse	TGGACCTGCAAGTTAAAATCCC
IL‐10	Forward	GACTTTAAGGGTTACCTGGGTTG
IL‐10	Reverse	TCACATGCGCCTTGATGTCTG
TGF‐β	Forward	GGCCAGATCCTGTCCAAGC
TGF‐β	Reverse	GTGGGTTTCCACCATTAGCAC
IL‐12	Forward	ACCCTGACCATCCAAGTCAAA
IL‐12	Reverse	TTGGCCTCGCATCTTAGAAAG
MCP‐1	Forward	CAGCCAGATGCAATCAATGCC
MCP‐1	Reverse	TGGAATCCTGAACCCACTTCT
GAPDH	Forward	TGACAACTTTGGTATCGTGGAAGG
GAPDH	Reverse	AGGCAGGGATGATGTTCTGGAGAG

### 2.9 Nitric Oxide (NO) Detection

After THP‐1 cells were collected, cells were added to RPMI 1640 medium with 10% exosome‐free serum and 200 ng/mL PMA for 24 h. Cells were then mixed with exosomes (final concentration: 10 μg/mL) for 72 h incubation. After that, the cell supernatant and cells were harvested and applied to detect the NO level using the Total Nitric Oxide Assay Kit (Beyotime, Shanghai, China) following the supplier's protocol. Optical density values at 540 nm were tested by a microplate reader (Bio‐Rad, USA).

### 2.10 Statistical Analysis

All data from at least three replicates were described as mean ± SD, and data analysis was completed by GraphPad Prism 8.4.2. Comparison among multiple groups was assessed by one‐way ANOVA with Tukey's post hoc test. *p* < 0.05 was identified as the criteria of statistically significance.

## 3 Results

### 3.1 Characterization of Exosomes Derived From P‐PRP and L‐PRP

**Figure 1:**
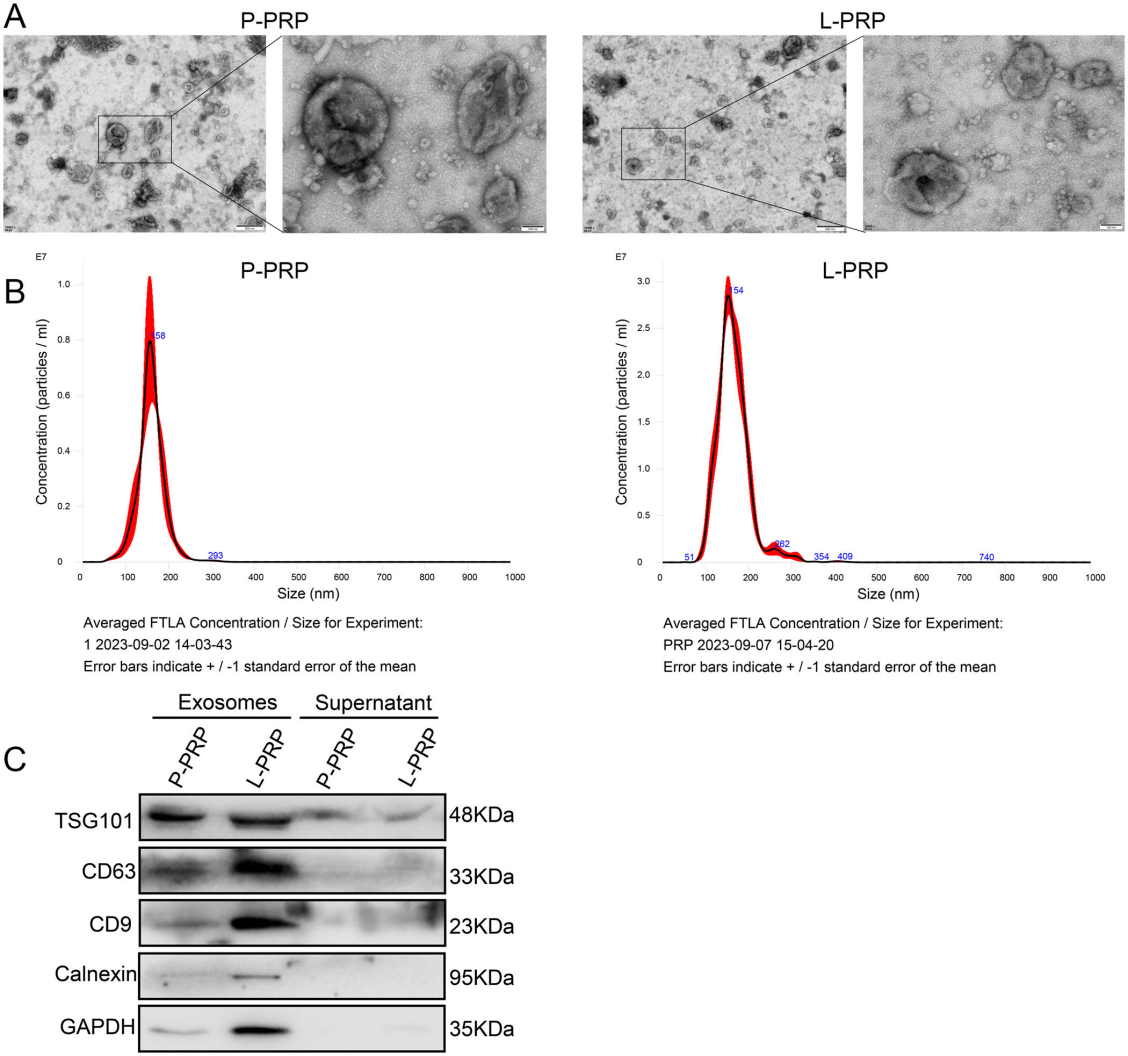
Characterization of exosomes derived from P‐PRP and L‐PRP. (A) The morphologies of exosomes derived from P‐PRP and L‐PRP were observed by TEM (left: ×10,000, right: ×40,000). (B) The particle size distribution of exosomes was determined by NTA. (C) Expression of TSG101, CD63, and CD9 in exosomes were assessed by the western blot.

To clarify the action of PRP‐derived exosomes on macrophages, the exosomes were first prepared from P‐PRP and L‐PRP, respectively. TEM results found that the extracted exosomes from P‐PRP and L‐PRP exhibited round or elliptical morphology with a complete double‐membrane structure (Figure 1A). Besides, the NTA results demonstrated that the diameters of exosomes from P‐PRP and L‐PRP were approximately 155 nm (Figure 1B). Furthermore, the presence of TSG101, CD63, and CD9 in the exosomes was confirmed (Figure 1C). Therefore, these results indicated that exosomes were successfully isolated from PRP.

### 3.2 Coculture of Exosomes From P‐PRP/L‐PRP and Macrophages

**Figure 2:**
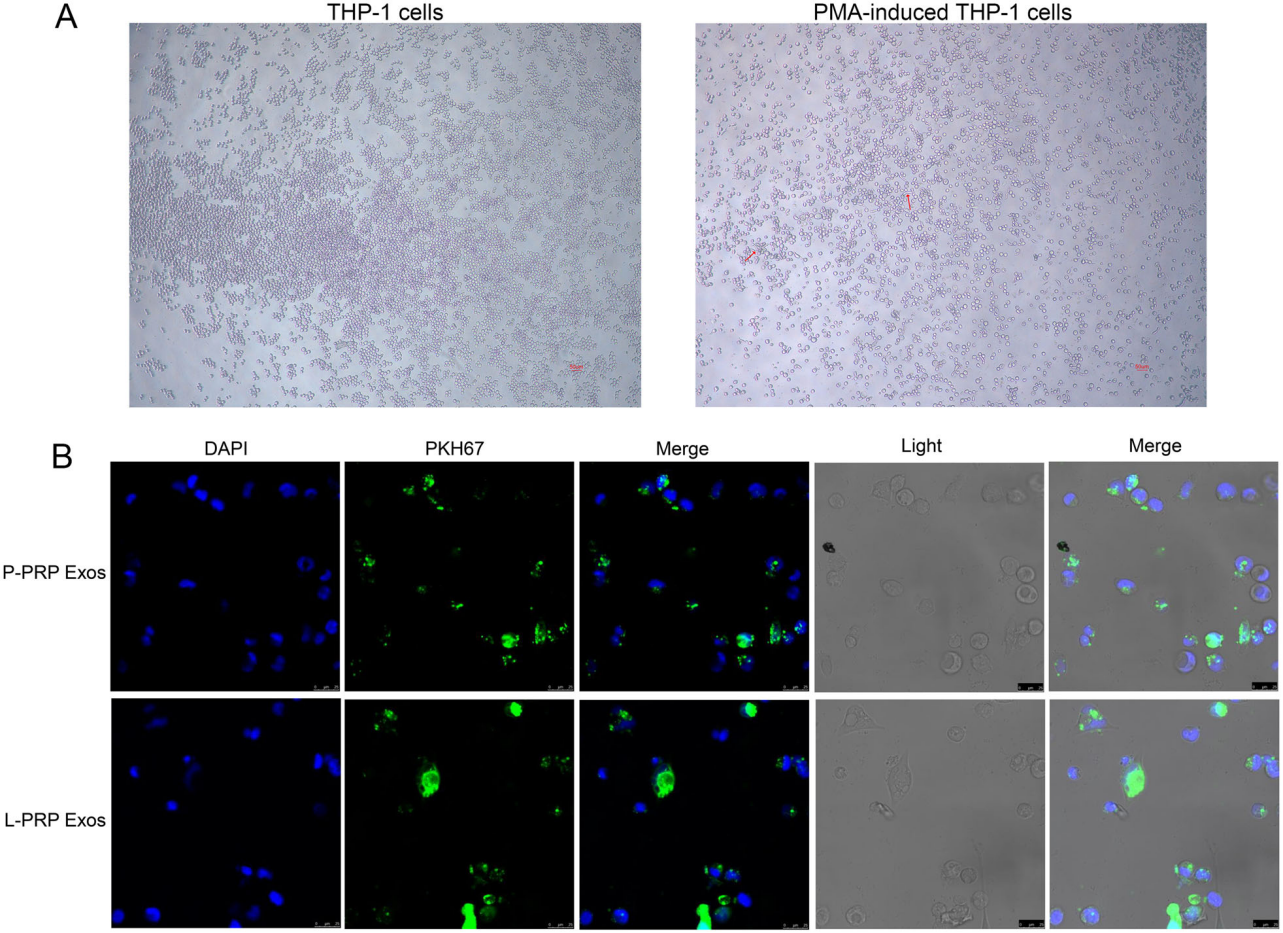
Exosomes from P‐PRP and L‐PRP were absorbed by macrophages. (A) Cell images of THP‐1 cells and macrophages induced using THP‐1 cells by PMA. (B) Internalization of PKH67 labeled exosomes isolated from P‐PRP and L‐PRP into macrophages after incubation of 24 h.

To explore whether macrophages could absorb the PRP‐derived exosomes, the macrophages were induced using THP‐1 cells by PMA. Results showed that THP‐1 cells appear to adhere to the wall and produce antennae after induced by PMA (Figure 2A), which indicated that macrophages were successfully induced. After that, internalization of PKH67‐labeled exosomes was observed after coculture of the exosomes and macrophages for 24 h. It was found that the exosomes from P‐PRP and L‐PRP were internalized by macrophages (Figure 2B). Thus, these results suggested that the exosomes from P‐PRP and L‐PRPR could be taken up by macrophages.

### 3.3 Exosomes From L‐PRP Restrained Viability and Induced Apoptosis of Macrophages

**Figure 3:**
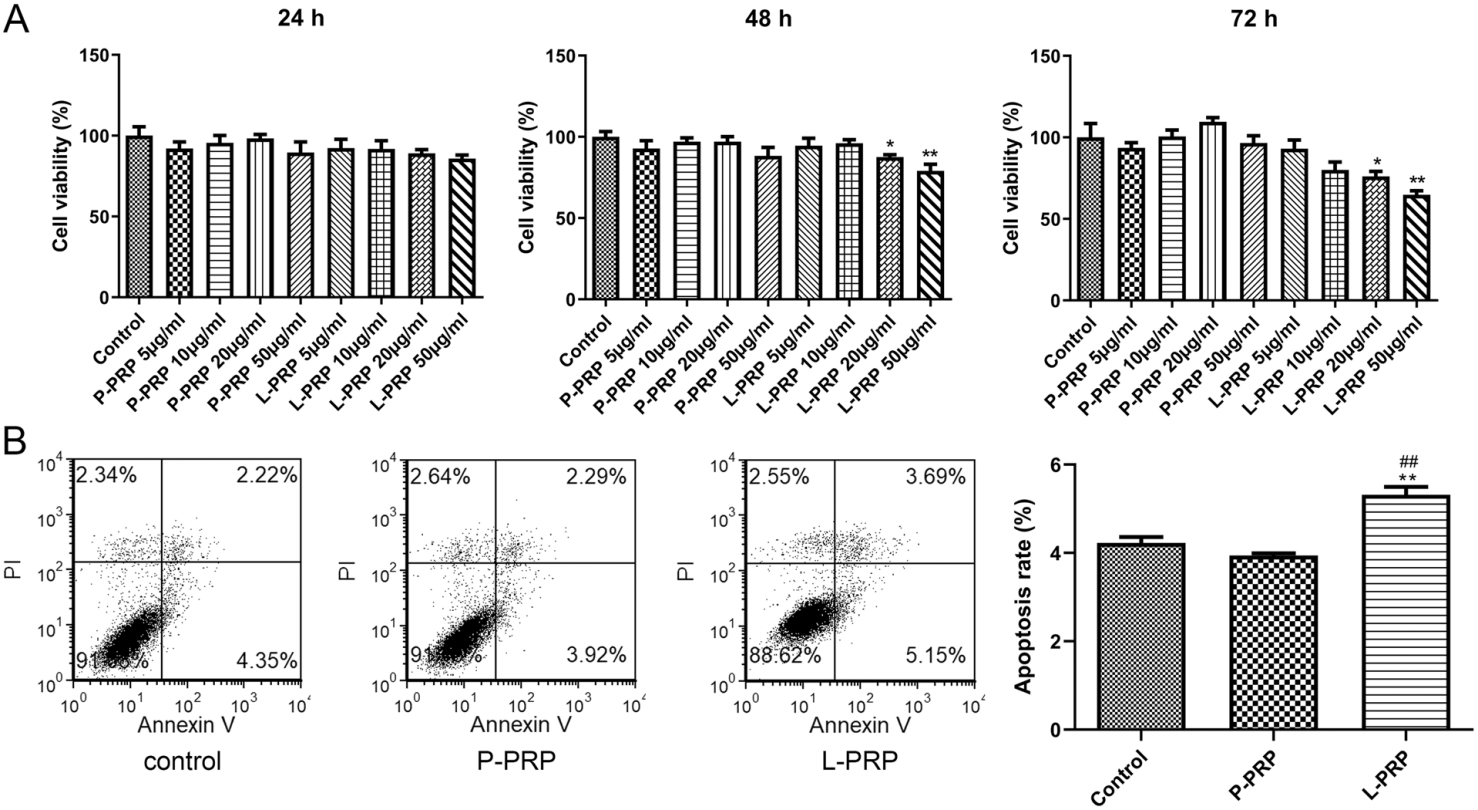
Exosomes from L‐PRP restrained proliferation and induced apoptosis of macrophages. (A) Cell viability of macrophages was assessed by CCK‐8 assay after treated by exosomes from P‐PRP and L‐PRP for 24, 48, and 72 h. (B) Cell apoptosis of macrophages was evaluated by flow cytometry after treated by exosomes from P‐PRP and L‐PRP for 72 h. *p < 0.05, **p < 0.01, vsersus control group; ^##^ *p* < 0.01 vsersus P‐PRP group.

To elaborate the action of PRP‐derived exosomes on macrophages, cell viability and apoptosis ability of macrophages were determined after treated by exosomes from P‐PRP and L‐PRP. Results showed that the exosomes from P‐PRP had no significant effect on the viability of macrophages at different times (24, 48, 72 h) and concentrations (5, 10, 20, 50 μg/mL) (Figure 3A). However, the exosomes from L‐PRP (20 and 50 μg/mL) significantly suppressed the viability of macrophages after treated for 48 and 72 h (*p* < 0.05, Figure 3A). According to this result, 20 μg/mL of exosomes with 72 h treatment was selected for subsequent experiments. Furthermore, flow cytometry results found that the exosomes from L‐PRP significantly promoted the apoptosis of macrophages (*p* < 0.01). In contrast, exosomes from P‐PRP did not affect the apoptosis of macrophages (Figure 3B). Hence, these results revealed that exosomes from L‐PRP restrained viability and induced apoptosis of macrophages.

### 3.4 Exosomes From P‐PRP Promoted M2 Polarization, and Exosomes From L‐PRP Promoted M1 Polarization

**Figure 4:**
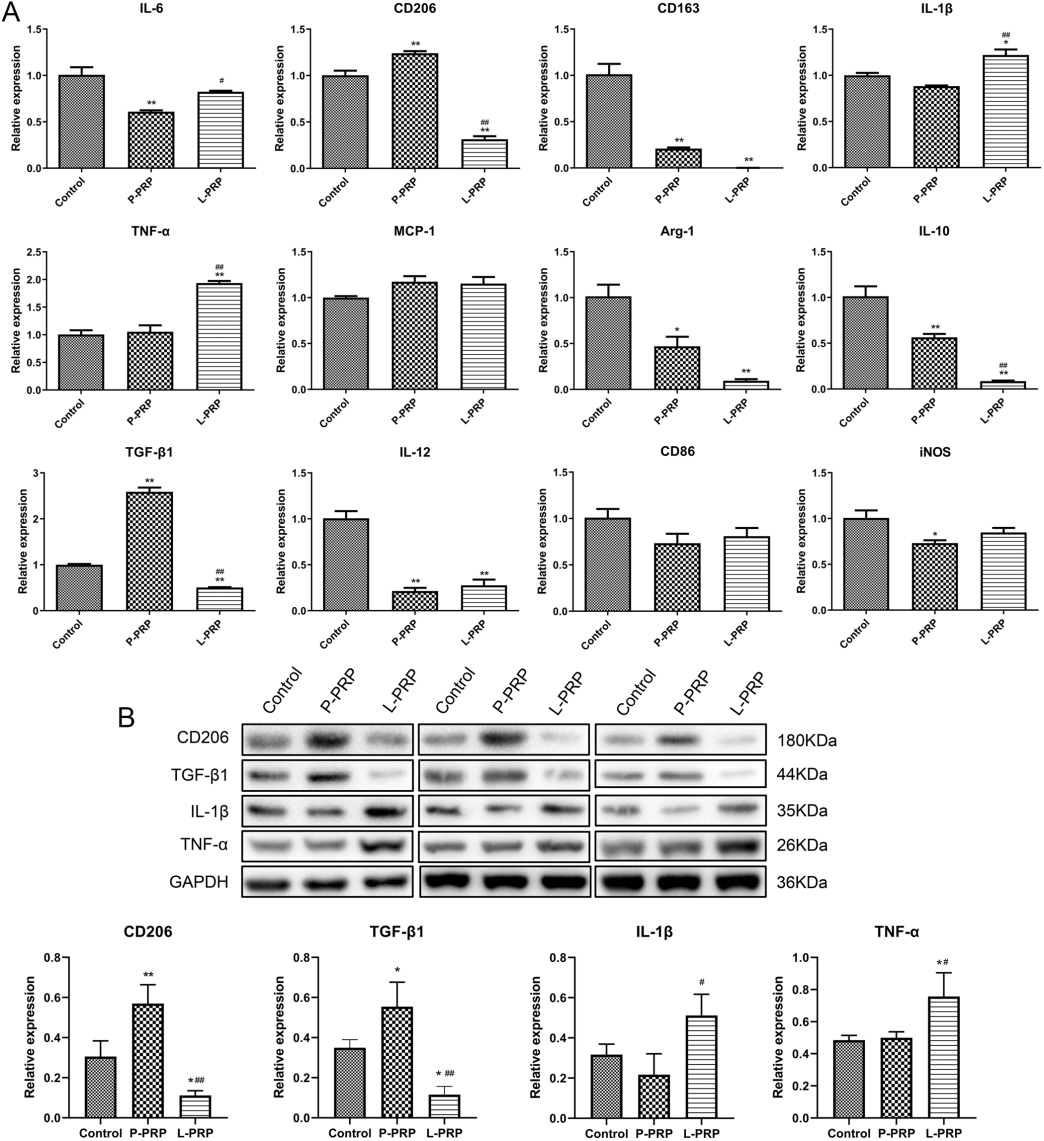
Exosomes from P‐PRP promoted M2 polarization, and exosomes from L‐PRP promoted M1 polarization. (A)The mRNA levels of IL‐6, IL‐1β, TNF‐α, IL‐12, CD86, iNOS, MCP‐1, CD206, CD163, TGF‐β1, IL‐10, and Arg‐1 in macrophages after treated by exosomes from P‐PRP and L‐PRP were determined using qRT‐PCR. (B) The protein levels of CD206, TGF‐β1, IL‐1β, and TNF‐α in macrophages were determined by western blot after treated by exosomes from P‐PRP and L‐PRP. *p < 0.05, **p < 0.01, versus control group; ^#^ *p* < 0.05, ^##^ *p* < 0.01 versus P‐PRP group.

To further investigate the action of PRP‐derived exosomes on macrophages, the role of exosomes in macrophage polarization was detected by testing the levels of M1 markers (IL‐6, IL‐1β, TNF‐α, IL‐12, CD86, iNOS, MCP‐1) and M2 markers (CD206, CD163, TGF‐β1, IL‐10, Arg‐1). Results showed that the exosomes from P‐PRP significantly enhanced the mRNA expressions of CD206 and TGF‐β1 (*p* < 0.05, Figure 4A). However, the exosomes from L‐PRP significantly elevated the mRNA expressions of IL‐1β and TNF‐α (*p* < 0.05, Figure 4A). Besides, western blot results found that the exosomes from P‐PRP increased the protein levels of CD206 and TGF‐β1, and the protein levels of IL‐1β and TNF‐α were elevated by exosomes from L‐PRP (*p *< 0.05, Figure 4B). These results indicated that exosomes from P‐PRP promoted M2 polarization, and exosomes from L‐PRP promoted M1 polarization.

### 3.5 Exosomes From ‐PRP Promoted No Generation of Macrophages

**Figure 5:**
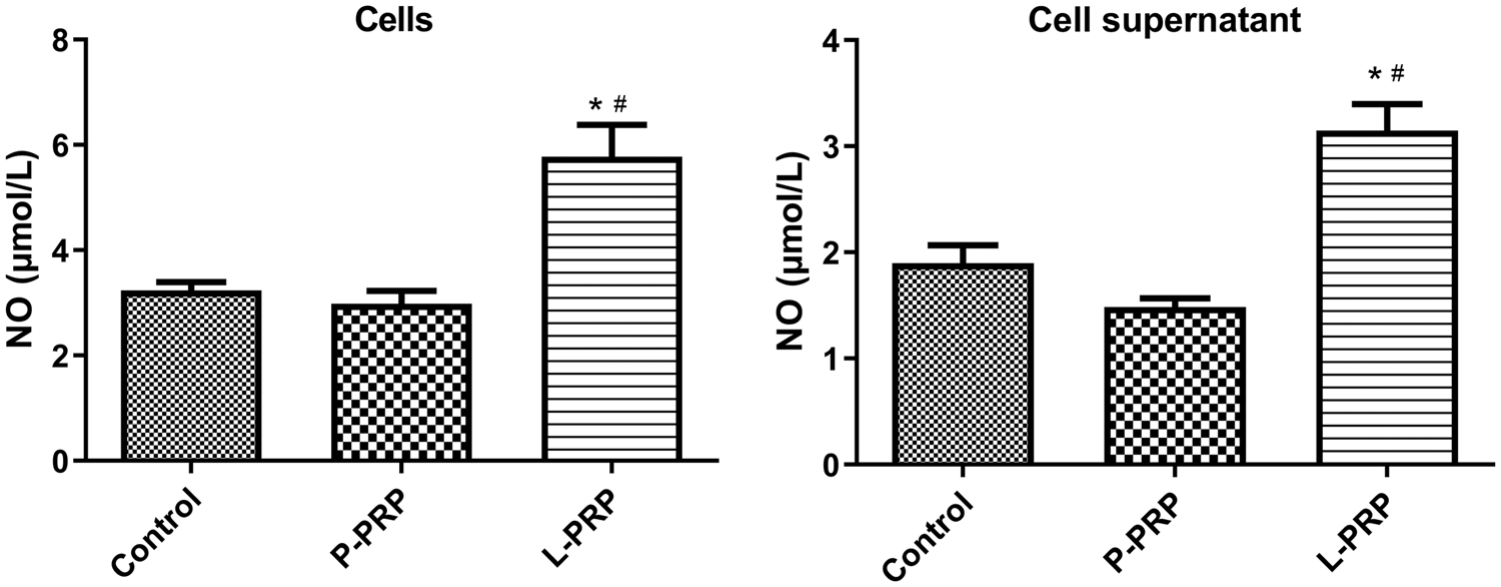
Exosomes from L‐PRP promoted NO generation of macrophages. The NO productions in macrophages and supernatant were determined after the NO production in macrophages and supernatant. *p < 0.05 versus control group; ^#^ *p* < 0.05 versus P‐PRP group.

To deeply explore the effect of PRP‐derived exosomes on macrophages, the NO generation of macrophages was determined after treated by exosomes. Results showed that the exosomes from P‐PRP had no significant effect on the NO production in macrophages and supernatant (Figure 5). Nevertheless, the exosomes from L‐PRP obviously strengthened the NO generation both in macrophages and supernatant (Figure 5). In brief, these results suggested that exosomes from L‐PRP promoted NO generation of macrophages.

## 4 Discussion

Chronic refractory wounds are those that cannot be repaired promptly and are accompanied by complicated pathogenesis, which pose great challenges to clinical health, reduce patients' quality of life, and increases the burden on families [6, 32]. PRP contains a high concentration of platelets, and it has significant potential in treating chronic wound healing [33, 34]. Recent studies reported that the exosomes isolated from PRP were more effective than PRP [35, 36]. However, the therapeutic potential of exosomes from PRP on chronic refractory wounds remained elusive. Hence, our study elucidated the role of exosomes from P‐PRP and L‐PRP on macrophages, which played critical roles in chronic refractory wounds. It found that exosomes from L‐PRP restrained viability, induced apoptosis and NO generation of macrophages, and promoted M1 polarization, while exosomes from P‐PRP increased M2 polarization.

Exosomes belong to extracellular vesicles (EVs) with a diameter of 40–160 nm [37]. It interprets a new way of intercellular communication, contributing to a wide range of biological processes in health and disease [38]. In general, exosomes in cells are extracted and purified by ultracentrifugation, which is regarded as the “gold standard” for exosome isolation [39]. Therefore, the exosomes in PRP were obtained using ultracentrifugation in this study. The diameters of exosomes from P‐PRP and L‐PRP were approximately 155 nm, which is within the reported diameter range [37]. Besides, the results showed that the exosomes from PRP were enriched in TSG101, CD63 and CD9. TSG101, CD63, and CD9 were generally considered to be the markers of exosomes [40, 41]. The above evidence indicated that exosomes were successfully isolated from P‐PRP and L‐PRP.

Macrophages are proven to exert a vital role in tissue remodeling of wounds [42, 43]. For instance, Oshima et al. [44] revealed that M2‐polarized macrophages mediate wound healing. Therefore, the effect of exosomes from PRP on chronic refractory wounds was detected by the response of macrophage to exosomes in this study. Human monocytic cell line THP‐1 is the most widely used model for primary macrophages because THP‐1 cells can acquire the macrophage‐like phenotype, which mimics primary human macrophages, after differentiation using PMA [45]. Therefore, macrophages were obtained in this study by inducing THP‐1 with PMA. After that, the effect of exosomes from PRP on macrophage proliferation and apoptosis was explored. The results found that exosomes from L‐PRP restrained viability and induced apoptosis of macrophages, while the exosomes from P‐PRP had no effect on macrophage viability and apoptosis. NO is a soluble endogenous gas with various biological functions [46]. In the immune response, macrophages can increase NO production, which is critical for pathogen killing [46]. In this study, the action of exosomes from PRP on the production of NO in macrophages was investigated. It was found that exosomes from L‐PRP promoted NO generation of macrophages, while exosomes from P‐PRP did not affect NO production of macrophages. The above results indicated that exosomes from L‐PRP possess more effective effects on macrophages than the exosomes from P‐PRP. Similarly, a previous study of Lin et al. proved that L‐PRP has better stimulating effects on tenocyte proliferation compared with the P‐PRP [47]. Another study expounded that L‐PRP could cause a greater acute inflammatory response than P‐PRP [48]. Furthermore, the effect of exosomes from PRP on macrophage polarization was determined. The results discovered that the exosomes from L‐PRP elevated the levels of IL‐1β and TNF‐α, while the exosomes from P‐PRP increased CD206 and TGF‐β1. IL‐1β and TNF‐α were the markers of M1 macrophages, and CD206 and TGF‐β1 were the markers of M2 macrophages [49, 50]. These results suggested that exosomes from P‐PRP promoted M2 polarization, and exosomes from L‐PRP promoted M1 polarization. Consistent with our study, Assirelli et al. demonstrated that L‐PRP induced a greater increase in the pro‐inflammatory factors IL‐1β than P‐PRP [51]. These findings suggested that the exosomes extracted from L‐PRP and P‐PRP might have significant functional consistency with L‐PRP and P‐PRP, respectively.

However, there are some limitations in this study. The therapeutic efficacy of the L‐PRP‐derived exosomes in chronic wounds should be further explored using an animal chronic wound model. Additionally, in the future, we also need to investigate whether the diseased macrophages from patients would respond in a similar fashion than healthy THP‐1 cells.

In conclusion, this study revealed that exosomes from L‐PRP restrained viability, induced apoptosis, and NO generation of macrophages and promoted M1 polarization, while exosomes from P‐PRP increased M2 polarization. The exosomes from L‐PRP presented a more effective effect on macrophages than that from P‐PRP, making it a promising strategy for chronic refractory wounds management.

## Author Contributions

X.L. and F.F.G. contributed to the conception, performed study concept, design and wrote the manuscript. J.H.D. acquired the data. J.S.L. and J.Z. helped with data analysis and revised the manuscript. M.F. analyzed the data. H.F. performed study concept, design and revised the manuscript. All authors contributed to the article and approved the final version.

## Ethics Statement

The study was approved by the Ethics Committee of the Second Affiliated Hospital of Guilin Medical College.

## Consent

All participants provided written informed consent.

## Conflicts of Interest Statement

The authors declare no conflicts of interest.

## Data Availability

The data that support the findings of this study are available on request from the corresponding author upon reasonable request.
